# SNARE Protein CfSec22 Mediates Vesicular Trafficking to Regulate Growth, Conidiogenesis, and Pathogenesis of *Ceratocystis fimbriata*

**DOI:** 10.3390/microorganisms13102305

**Published:** 2025-10-05

**Authors:** Changgen Li, Yiming Wang, Xiaoying Cao, Kailun Lu, Lianwei Li, Jihong Jiang

**Affiliations:** 1 The Key Laboratory of Biotechnology for Medicinal and Edible Plant Resources of Jiangsu Province, School of Life Sciences, Jiangsu Normal University, Xuzhou 221116, China; 1020200018@jsnu.edu.cn (C.L.); 1020210013@jsnu.edu.cn (Y.W.); cxy4868@jsnu.edu.cn (X.C.); 1020220017@jsnu.edu.cn (K.L.); 2 School of Modern Agricultural and Forestry, Yancheng Agricultural College, Yancheng 224051, China

**Keywords:** *Ceratocystis fimbriata*, pathogenesis, SNARE protein, vesicular transport

## Abstract

Soluble N-ethylmaleimide-sensitive factor attachment protein receptor (SNARE) proteins play evolutionarily conserved roles in intracellular vesicle trafficking and membrane fusion across eukaryotes. In pathogenic fungi, various SNARE homologs have been shown to critically regulate host infection processes. Here, we characterize the functional roles of CfSec22 in the sweet potato black rot pathogen *Ceratocystis fimbriata*. Phylogenetic and domain analyses demonstrate that CfSec22 shares homology with Sec22 proteins from *Saccharomyces cerevisiae* (ScSec22), *Magnaporthe oryzae* (MoSec22), and other fungi, containing both the characteristic Longin homology domain and V-SNARE domain. Functional studies reveal that CfSec22 regulates growth, conidiation, and virulence of *C. fimbriata*. Deletion of *CfSEC22* resulted in abnormal vacuole morphology and impaired endocytosis. The Δ*Cfsec22* mutant displayed heightened sensitivity to diverse stress conditions: oxidative, endoplasmic reticulum, and cell wall stressors. Subcellular localization studies confirmed the endoplasmic reticulum residence of CfSec22. Finally, we established that CfSec22 regulates the secretion of virulence-associated proteins and is required for the induction of ipomeamarone in infected sweet potato tissues. Together, our findings demonstrate that CfSec22-mediated vesicle trafficking serves as a critical regulatory mechanism supporting growth, conidiogenesis, and pathogenicity in *C. fimbriata*.

## 1. Introduction

*Ceratocystis fimbriata* is the causal agent of sweet potato black rot, a devastating fungal disease that significantly reduces the yield and quality of sweet potato crops worldwide during both cultivation and storage [1,2,3]. The pathogen is dispersed through various means, including soil, infected storage roots, and seedlings [4,5,6]. *C. fimbriata* infects multiple parts of sweet potato, typically causing gray or brown necrotic lesions on the surface of storage roots [7,8,9]. When young leaves are infected, the pathogen can directly penetrate the epidermis, leading to the formation of visible lesions. The peltate glandular trichomes, which provide nourishment and anchorage for *C. fimbriata*, serve as one of the invasion routes into leaf tissues [10]. In storage roots, wounds—particularly those incurred during harvest—act as the primary entry point for the pathogen, often resulting in late-stage morbidity [11]. In response to *C. fimbriata* invasion, sweet potato tissues produce furanoid terpenoids such as ipomeamarone [12] to resist pathogen infection [13]. Accidental consumption of infected roots can cause hepatic and respiratory toxicity, including pneumonia, pulmonary edema, and even fatal zoonotic reactions [14,15]. Therefore, effective management of sweet potato black rot is critical for global food security. Despite its agricultural significance, the pathogenic mechanism of *C. fimbriata* remains poorly understood, particularly the molecular regulatory mechanism of pathogenicity-associated genes.

Vesicle-mediated membrane trafficking [16] is a highly conserved transport pathway responsible for the precise shuttling of cargo between the endoplasmic reticulum (ER), Golgi apparatus, lysosomes or vacuoles, and other cellular compartments. This pathway underpins critical cellular processes, including intracellular signaling, hormone secretion, and exosome release [17]. Multiple protein families regulate vesicular trafficking, including SNARE proteins, SM (Sec1/Munc18) proteins, small GTPases, and HOPS (homotypic fusion and protein sorting) complexes [18,19]. Among these, the SNARE protein family plays a central role in mediating membrane fusion during secretory and exocytotic pathways [20,21]. SNARE proteins directly facilitate vesicle docking and fusion with target membranes. Structurally, they contain a C-terminal transmembrane domain and a conserved SNARE motif spanning 60–70 residues [22]. In filamentous fungi such as *Aspergillus oryzae* [23] and *Fusarium graminearum* [24], 21 putative SNARE proteins have been identified, each playing a crucial role in vesicle trafficking regulation.

Sec22, a member of the SNARE protein family, primarily mediates anterograde and retrograde trafficking between the ER and Golgi apparatus [25]. Previous studies showed that Sec22 is localized in the ER in *F. graminearum* [26]. Its homologs are implicated in diverse biological processes across organisms. In *Saccharomyces cerevisiae*, deletion of *ScSEC22* reduces spore production, disrupts budding, and diminishes stress resistance [27]. Similarly, Sec22 is essential for growth, development, and pathogenicity in several plant pathogenic fungi. In *Magnaporthe oryzae*, disruption of *MoSEC22* impairs hyphal growth, sporulation, and virulence while also compromising cell wall integrity and reducing extracellular enzyme production (e.g., peroxidase and laccase) [28]. In *Fusarium graminearum*, FgSec22 is required for the vegetative growth, pathogenesis, and deoxynivalenol (DON) biosynthesis [26]. *Verticillium dahlia* lacking *VdSEC22* exhibits attenuated virulence and impaired secretion of carbohydrate-hydrolyzing enzymes [29]. In *Colletotrichum orbiculare*, the ∆*Cosec22* mutant shows defective effector transmission [30]. In *Sordaria macrospora*, *SmSEC22* deletion leads to reduced ascospore production, impaired pigmentation, and germination defects [31]. Additionally, Sec22 regulates autophagy and cesium ion uptake [32]. Collectively, these findings underscore the essential role of Sec22 in diverse cellular processes across eukaryotic organisms.

Sweet potato is a globally important tuber crop cultivated in over 120 countries and regions [33,34]. However, its production faces serious threats from sweet potato black rot, a devastating disease caused by the fungal pathogen *Ceratocystis fimbriata* that affects both pre- and post-harvest stages [35]. This pathogen demonstrates wide geographical distribution, prolonged infectivity periods, and multiple transmission routes [7]. Annual yield losses attributed to sweet potato black rot typically range from 5% to 10%, but can reach 20–50% in severe outbreaks [14,36]. Investigation of virulence-associated genes in phytopathogenic fungi can provide fundamental insights into pathogenesis and inform novel control approaches [37,38,39]. Understanding the molecular mechanisms underlying *C. fimbriata* pathogenicity is crucial for developing effective disease management strategies. Recent studies have emphasized the critical role of vesicular transport proteins in fungal pathogenicity [18]. Nevertheless, research specifically addressing sweet potato black rot remains limited. To address this knowledge gap, we investigated the biological function of CfSec22, a SNARE protein in *C. fimbriata*, during sweet potato black rot development. Our results demonstrate that CfSec22 regulates morphogenesis and pathogenicity of *C. fimbriata* by modulating processes including endocytosis, reactive oxygen species (ROS) production, ER stress responses, and cell wall integrity maintenance. These insights advance our understanding of the infection mechanisms of *C. fimbriata*.

## 2. Materials and Methods

### 2.1. Fungal Strains and Culture Conditions

The study employed three *C. fimbriata* strains: wild-type BMPZ13 [10], a Δ*Cfsec22* mutant, and a complemented strain. All strains were cultured on complete medium (CM) at a temperature of 27 °C for 5 days. For stress response assays, CM plates were supplemented with various stress agents, including 0.3 M NaCl, 0.3 M KCl, 0.4 M sorbitol, 400 μg/mL Congo Red, 50 mM DTT, or 1 mM H_2_O_2_ [38]. A 2 mm mycelial plug was inoculated onto each plate and incubated at 27 °C for 5 days, with colony diameters measured to assess stress tolerance (two perpendicular measurements).

### 2.2. Bioinformatics Analysis

Protein sequences were obtained from NCBI (https://www.ncbi.nlm.nih.gov/protein/PHH55958.1?report=genpept, accessed on 1 May 2025). Using Saccharomyces cerevisiae Sec22 as a query, we identified homologs in *C. fimbriata*. Protein structure prediction was performed using Phyre2 (http://www.sbg.bio.ic.ac.uk/phyre2/html/page.cgi?id=index, accessed on 1 May 2025), while sequence alignment used DNAMAN9. The Golgi dynamics (GOLD) domain was analyzed using WebLogo (https://weblogo.threeplusone.com/create.cgi, accessed on 1 May 2025). A phylogenetic tree based on the homologous protein sequences was reconstructed using MEGA 7.0 with the maximum-likelihood method (mL). A total of 1000 bootstrap replicates were executed to calculate the robustness of the mL tree.

### 2.3. Gene Knockout and Complementation

To investigate CfSec22 function in *C. fimbriata*, we generated ∆*Cfsec22* mutant and complemented strains ∆*Cfsec22/CfSEC22*. A standard one-step gene replacement strategy was employed to construct deletion mutants of the *CfSEC22* gene (Table S1 and Figure S1) [40]. Transformants were screened on culture medium plates with the addition of hygromycin B, and verified by PCR and Southern blot (Figure S2A,B). For complementation, the *CfSEC22* gene with native promoter was cloned into pYF11 and transformed into Δ*Cfsec22*.

### 2.4. Pathogenicity Assays

To investigate the role of CfSec22 in the pathogenesis of sweet potato, a series of pathogenicity assays were performed. Sweet potato (Yan 25) [41] was cultivated at Jiangsu Normal University for pathogenicity evaluation. Three distinct infection assays were conducted to assess fungal virulence. For storage root slice infections, 2 mm mycelial plugs from test strains were inoculated onto freshly prepared root slices and incubated at 27 °C for 7 days. Whole storage root infections were performed by creating artificial wounds that were inoculated with 10 μL of spore suspension (10^6^ spores/mL), with inoculated roots maintained under dark conditions at high humidity for 15 days. Seedling infections were carried out by soaking cuttings in spore suspension for 30 min prior to planting in nutrient soil, with disease progression monitored over 15 days of growth at 27 °C.

### 2.5. Subcellular Localization

The RFP-HDEL construct in pYF11 was transformed into complemented strains via protoplast transformation. Transformants were selected with G418 and observed under a fluorescence microscope (Leica dm5000b, Ernst-Leit z-Strasse 17-37 D-35578 Wetzlar).

### 2.6. Endocytosis Assay

Wild-type and mutant strains were cultured in CM liquid medium at 27 °C for 36 h, stained with 0.8 μM FM4–64, washed with ddH_2_O, and imaged by a fluorescence microscope.

### 2.7. Ipomeamarone Detection

Ipomeamarone, the principal toxic metabolite produced in sweet potato during black rot infection [15], serves as a key phytoalexin in the plant’s defense response against pathogens [42]. We quantified ipomeamarone levels in infected sweet potato samples using GC-MS analysis. Due to the unavailability of an ipomeamarone standard, compound identification was performed by matching retention times and mass spectra with the Gas Chromatography Library reference. An amount of 100 g of infected storage roots was homogenized and extracted with methanol. After concentration and dichloromethane partitioning, samples were analyzed by GC-MS HP-5 column with helium carrier gas (1.2 mL/min). The temperature program was 40 °C (3 min) → 280 °C (10 °C/min) → hold 10 min.

### 2.8. Gene Expression Analysis

Total RNA was extracted using a Fungal Total RNA Isolation Kit (Sangon Biotech, Shanghai, China). cDNA was synthesized with HiScript II Q RT Supermix, and qPCR was performed using ChamQ SYBR qPCR Master Mix (Vazyme, Nanjing, China) and specific primers (Table S1). Relative expression was calculated by the 2^−ΔΔCt^ method using ACTIN as a reference. The cycling program was as follows: 95 °C for 30 s, followed by 40 cycles of 95 °C for 10 s and 60 °C for 30 s. All qRT-PCR assays were performed in triplicate and repeated three times.

### 2.9. Secretome Analysis

Mycelia were cultured in sweet potato juice medium at 27 °C for 7 days. After centrifugation (10,000 rpm, 4 °C) and 0.45 μm filtration, proteins were concentrated by freeze-drying. LC-MS/MS analysis was performed by Majorbio (Shanghai), with raw data deposited at CNCB China National Center for Bioinformation (OMIX010249).

### 2.10. Statistical Analysis

All experiments in this study were performed with a minimum of three biological replicates per trial and repeated at least three times. Statistical analyses and plotting were performed using GraphPad Prism 7.0 (GraphPad Software Inc., Boston, MA, USA). The values were presented as the mean ± standard deviations. Error bars represent standard deviations. Statistically significant differences among the mean values were assessed by Student’s *t*-test for two groups of data or by one-way ANOVA (Tukey’s post hoc test) for multiple comparisons tests.

## 3. Results

### 3.1. Characteristic of CfSec22

BLASTp analysis using Sec22 proteins from *S. cerevisiae* as a query revealed HPP55958 as the *C. fimbriata* homolog, containing conserved Longin and V-SNARE coiled-coil domains indicative of its potential roles in membrane fusion, endocytosis, and secretory processes. Three-dimensional modeling using Phyre2 demonstrated structural similarity between CfSec22 and ScSec22 (Figure S1A), which was further supported by WebLogo analysis of the V-SNARE domain across species (Figure S1B). Sequence alignment showed CfSec22 shares high identity with homologs from *S. cerevisiae*, *Verticillium dahliae*, *F. graminearum*, *Pyricularia oryzae*, and *Sclerotinia sclerotiorum* (47.20%, 70.56%, 77.21%, 75.46%, and 73.49%, respectively). Phylogenetic analysis of Sec22 revealed that *C. fimbriata*, *Thielaviopsis punctulata*, and *Colletotrichum trifolii* cluster together (Figure S1C). These findings provide evolutionary context for the functional characterization of CfSec22.

### 3.2. CfSec22 Is Involved in Growth and Sporulation

**Figure 1:**
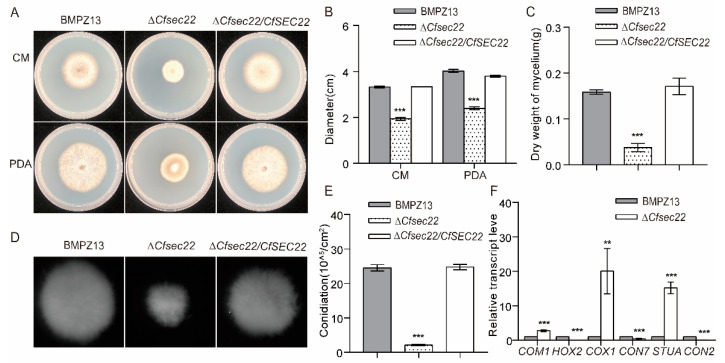
Colony morphology observation and sporulation of CfSec22. (**A**,**B**) Growth of strains on different media. The test strains were inoculated into CM and PDA medium and cultured at 27 °C for 5 days to measure their diameter. (**C**) Dry weight weighing of mycelium. The test strains were inoculated into CM liquid culture medium at 27 °C (150 rpm) for 5 days. The bacterial bodies were dried and weighed at 60 °C. (**D**) Observation of mycelium morphology. The test strains were inoculated into CM liquid culture medium at 27 °C (150 rpm) for 1 day to observe the morphology and size of the mycelium. (**E**) Spore yield statistics. The test strains were inoculated into CM and cultured at 27 °C for 5 days to calculate the number of spores. (**F**) Sporulation-related gene expression assays. Data are presented as the mean ± SD from three independent experiments. A one-way ANOVA was performed for multiple comparisons, whereas the *t*-test was completed for the comparison of two experimental groups: ** *p* < 0.01, and *** *p* < 0.001.

Phenotypic analysis revealed striking growth defects in the ∆*Cfsec22* mutant, with colony diameters reduced by 41.57% on CM and 40.55% on PAD compared to wild-type and complemented strains (Figure 1A,B). Moreover, complementation fully restored growth rates. While mycelial morphology appeared normal in liquid CM culture, ∆*Cfsec22* showed significantly reduced mycelial diameter and biomass accumulation (Figure 1C,D). Most notably, sporulation capacity was severely impaired (91.48% reduction) (Figure 1E), accompanied by differential expression of sporulation-related genes (Figure 1F). Collectively, these results suggest that CfSec22 plays a critical role in growth and development in *C. fimbriata*.

### 3.3. CfSec22 Is Essential for Full Pathogenicity in C. fimbriata

**Figure 2:**
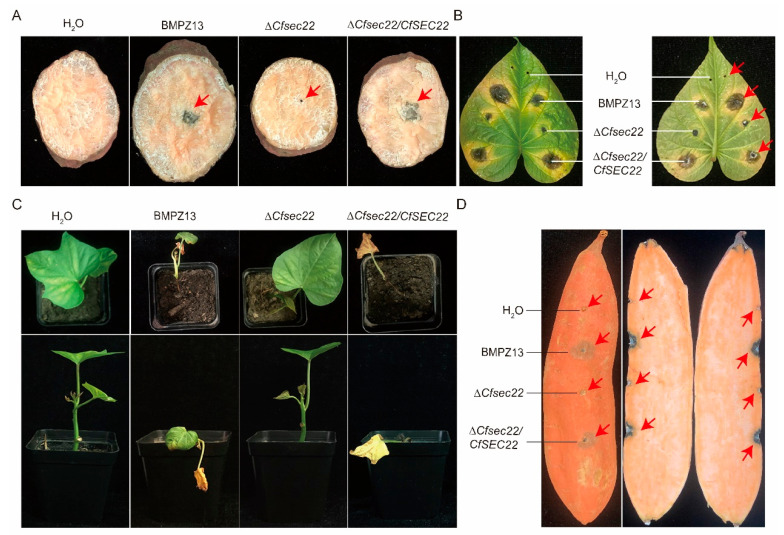
Toxicity determination of CfSec22 on sweet potato. (**A**) Toxicity test of mushroom cake on sweet potato storage root slices. Mushroom cake was inoculated on sweet potato storage root slices at 27 °C, and the hyphal colonization was observed after 7 days. (**B**) Toxicity of spore suspension on detached leaves of sweet potato. The leaves of sweet potato were drilled with holes, inoculated with spore suspension, and observed for the infection at 27 °C. (**C**) Experiment on infection of sweet potato cutting seedlings. Sweet potato cutting seedlings were soaked for 30 min, then cut in nutrient soil, and the infection was observed at 27 °C. (**D**) Experiment on infection of sweet potato storage roots. Sweet potato storage roots were drilled with holes, inoculated with spore suspension, moisturized, and observed for infection at 27 °C.

On storage root slices, the mutant failed to produce characteristic black rot lesions observed with wild-type and complemented strains (Figure 2A). Leaf inoculation assays showed significantly reduced lesion development (Figure 2B), while seedling infections demonstrated complete loss of virulence of Δ*Cfsec22*-inoculated plants, which remained healthy compared to wild-type-inoculated plants showing stem rot, lodging, and wilting (Figure 2C). Storage root inoculation confirmed attenuated lesion formation by Δ*Cfsec22* (Figure 2D). These results establish CfSec22 as essential for full pathogenicity in *C. fimbriata*.

### 3.4. CfSec22 Regulates Stress Response

**Figure 3:**
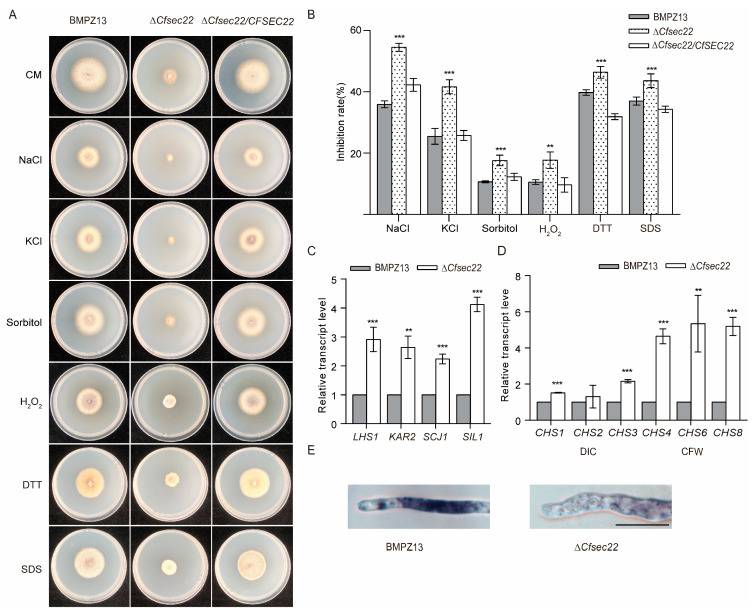
Stress determination of CfSec22 on the external environment. (**A**,**B**) Growth of the strain in the medium containing NaCl, KCl, sorbitol, H_2_O_2_, DTT, and SDS. (**C**) ER stress-related gene expression detection. (**D**) The expression level of chitin synthesis-related enzymes was detected by qRT-PCR. (**E**) The content of reactive oxygen species was observed by NBT staining. Scale bar, 10 μm. Data are presented as the mean ± SD from three independent experiments. A one-way ANOVA was performed for multiple comparisons: ** *p* < 0.01, and *** *p* < 0.001. Scale bar, 10 μm.

The mutant exhibited heightened sensitivity to osmotic (NaCl, KCl, sorbitol) and cell wall stressors (SDS) (Figure 3A,B), with altered expression of chitin synthase genes (Figure 3D). The mutant also exhibited heightened sensitivity to ER stressor (DTT) (Figure 3A,B) and upregulation of ER chaperone-encoding genes (Figure 3C). In addition, oxidative stress susceptibility was evaluated using H_2_O_2_ treatment. The Δ*Cfsec22* mutant showed significantly greater growth inhibition than control strains (Figure 3A,B), indicating compromised oxidative stress defense mechanisms. This phenotype was corroborated by NBT (tetranitroblue tetrazolium chloride) staining, which revealed reduced reactive oxygen species (ROS) accumulation at hyphal tips (Figure 3E). These findings position CfSec22 as a multifunctional regulator of stress adaptation in *C. fimbriata*.

### 3.5. The Absence of CfSEC22 Affects the Ipomeamarone Production in Sweet Potato

**Figure 4:**
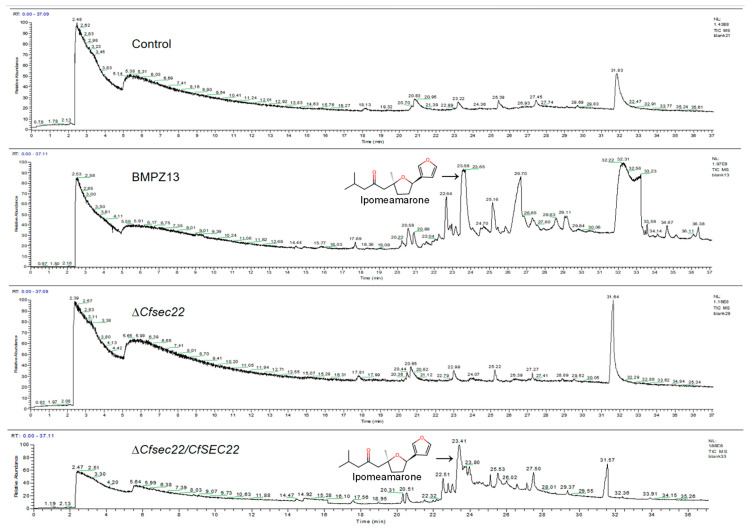
Representative GC-MS total ion chromatogram of ipomeamarone.

The analysis revealed a peak at 23.65 min corresponding to ipomeamarone in wild-type BMPZ13-infected samples, while this compound was undetectable in both uninfected controls and Δ*Cfsec22* mutant-infected samples (Figure 4). These results suggest that CfSec22 may play a regulatory role in ipomeamarone biosynthesis during *C. fimbriata*-sweet potato storage root interactions.

### 3.6. CfSEC22 Is Located in the ER and Affects Endocytosis

**Figure 5:**
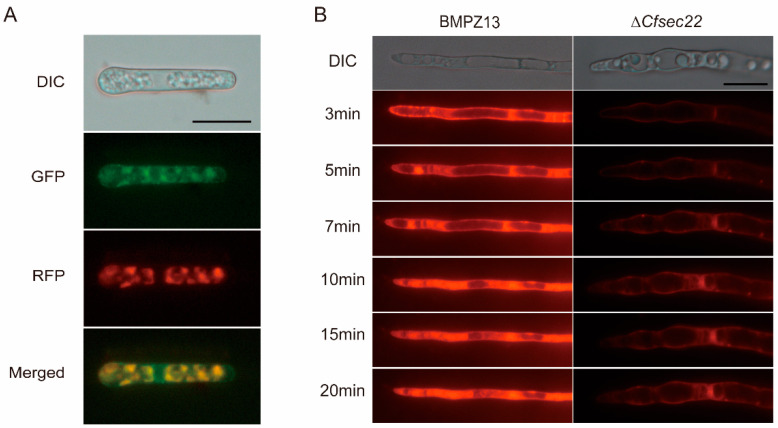
Localization and endocytosis. (**A**) Subcellular localization of CfSec22 protein. (**B**) FM4–64 staining. The endocytosis of FM4–64 was observed using a fluorescence microscope after samples were stained with 8 mM FM4–64. Scale bar, 10 μm.

To determine CfSec22 localization in *C. fimbriata*, we transformed an ER marker, RFP-HDEL, into the complemented strain. The red and green fluorescent signals overlapped completely, indicating that CfSec22 is localized to the intracellular ER (Figure 5A). As a vesicular transport protein, Sec22 is involved in transportation and secretion [25]. We investigated CfSec22’s role in endocytosis using FM4–64 uptake assays. The Δ*Cfsec22* mutant exhibited significantly delayed endocytosis, requiring nearly 10 min for dye internalization compared to 3 min in wild-type hyphae (Figure 5B). This phenotype suggests that CfSec22 plays a role in the endocytosis process in *C. fimbriata*.

### 3.7. Proteomic Analysis of Secretory Defects of ΔCfsec22 Mutant

**Figure 6:**
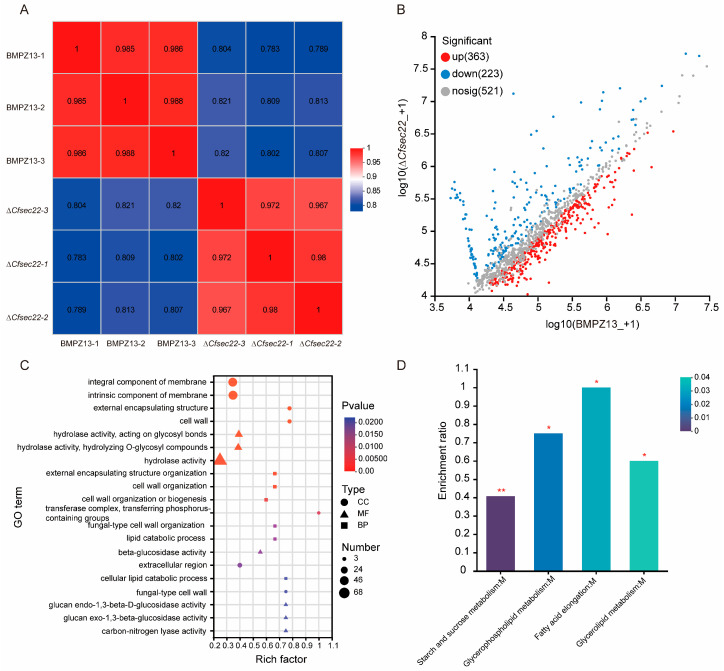
Secretory proteome analysis. (**A**) Correlation between samples. (**B**) Differential protein scatter plot. (**C**) GO enrichment analysis. (**D**) KEGG enrichment analysis, The X is KEGG pathway, and the Y is enrichment rate. (* *p* < 0.05, ** *p* < 0.01).

Comprehensive iTRAQ-based quantitative proteomic profiling identified substantial differences in extracellular secretory protein content between wild-type and Δ*Cfsec22* strains, with 586 proteins showing differential levels of secretion (363 upregulated and 223 downregulated; Figure 6A,B). Gene ontology enrichment analysis of the downregulated protein subset revealed significant associations with several critical biological processes, including external encapsulating structure organization, cell wall biogenesis, and lipid catabolic processes. At the cellular component level, these proteins were predominantly localized to membrane systems, cell wall structures, and transferase complexes. Molecular function analysis highlighted significant enrichment of hydrolase activity, β-glucosidase activity, and carbon–nitrogen lyase activity. Of particular note, the downregulated protein set included 39 proteins involved in exocrine secretion and 18 hydrolases (Figure 6C). KEGG pathway analysis further identified 71 significantly altered metabolic pathways, with four metabolism-related pathways showing particularly pronounced changes (Figure 6D). Examination of the 10 most substantially downregulated extracellular proteins revealed that 9 contained characteristic signal peptides (Table 1), suggesting potential roles in secretory processes.

### 3.8. Functional Validation of Candidate Secretory Proteins

**Figure 7:**
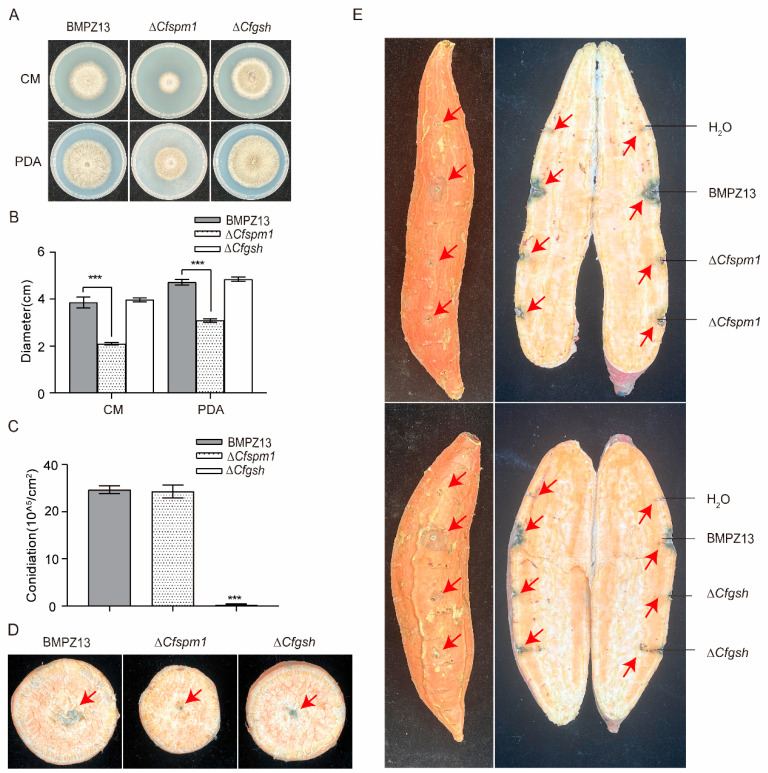
Colony morphology observation and sporulation of CfSpm1 and Cfgsh. (**A**,**B**) Growth of strains on different media. The test strains were inoculated into CM and PDA medium and cultured at 27 °C for 5 days to measure their diameter. (**C**) Spore yield statistics. The test strains were inoculated into CM and cultured at 27 °C for 5 days to calculate the number of spores. (**D**) Toxicity test of mushroom cake on sweet potato storage root slices. Mushroom cake was inoculated on sweet potato storage root slices at 27 °C, and the hyphal colonization was observed after 7 days. (**E**) Experiment on infection of sweet potato storage roots. Sweet potato storage roots were drilled with holes, inoculated with spore suspension, moisturized, and observed for infection at 27 °C. Data are presented as the mean ± SD from three independent experiments. A one-way ANOVA was performed for multiple comparisons, whereas the *t*-test was completed for the comparison of two experimental groups: *** *p* < 0.001.

Based on the proteomic findings, we selected two candidate secretory proteins, CfSpm1 (PHH54118.1) and CfGsh (PHH55903.1), for functional characterization through gene knockout studies (Figures S3–S5). The ∆*Cfspm1* mutant displayed a markedly reduced growth rate while maintaining normal sporulation capacity, whereas ∆*Cfgsh* exhibited wild-type growth characteristics but significantly impaired sporulation (Figure 7A–C). Pathogenicity assays on sweet potato storage roots demonstrated that ∆*Cfspm1* and ∆*Cfgsh* showed attenuated virulence compared to the wild-type (Figure 7D,E), providing compelling evidence for their involvement in *C. fimbriata* pathogenesis. These results validate the proteomic predictions and establish the functional importance of these candidate secretory proteins in fungal virulence mechanisms.

## 4. Discussion

SNARE proteins play a crucial role in the growth, development, sporulation, and pathogenicity of fungal pathogens [18]. Our study reveals that CfSec22 in *C. fimbriata* shares structural and functional conservation with Sec22 homologs in other fungi, containing characteristic Longin and V-SNARE coiled-coil domains. Similarly to MoSec22 [28], FgSec22 [26], and VdSec22 [43], CfSec22 plays critical roles in fungal growth and virulence. The Δ*Cfsec22* mutant exhibited pleiotropic defects, including reduced growth rate, impaired sporulation, smaller mycelial pellets, and attenuated pathogenicity across all tested infection models, including storage roots, slices, leaves, and seedlings. CfSec22 displays a typical V-SNARE cooled-coil topology structure, which was further confirmed through modeling using the WebLogo and Phyre2 online tools. These findings confirmed the evolutionary conservation of SNARE protein functions in filamentous fungi.

The endoplasmic reticulum-localized CfSec22 appears to regulate multiple cellular processes through vesicular trafficking. In eukaryotic cells, endocytosis mediates nutrient uptake, protein/lipid transport, and signal transduction [44,45]. The delayed FM4–64 internalization kinetics (10 min in mutant vs. 3 min in wild-type) and aberrant vacuolar morphology observed in Δ*Cfsec22* suggest profound defects in endocytic pathways. These results align with established models of SNARE-mediated vesicle cycling [44,45,46] and mirror observations in *M. oryzae*, where MoVam7, MoSyn8, and MoSec22 [47,48] mediate intracellular trafficking to regulate hyphal growth, sporulation, and pathogenicity. The pleiotropic nature of Δ*Cfsec22* phenotypes likely stems from disruption of multiple membrane trafficking routes converging on the ER–Golgi interface.

Our stress response analyses revealed that Δ*Cfsec22* displays heightened sensitivity to multiple environmental challenges. The mutant showed increased vulnerability to cell wall, osmotic, ER, and oxidative stresses, likely due to impaired vesicle-mediated stress adaptation mechanisms. The vacuole is a dynamic structure, and intracellular vacuoles have been found to be associated with different extracellular conditions [49,50]. Vesicle-mediated dynamic changes regulate the surface-to-volume ratio, thereby maintaining tension in the vacuolar boundary membrane [51]. These findings align with observations in other plant pathogenic fungi, where disruption of vesicular transport components consistently leads to abnormal stress responses. In both *M. oryzae* and *F. graminearum*, various proteins involved in vesicle trafficking—including MoHse1, MoVps27, FgVam7, and FgVps27—have been shown to mediate responses to cell wall and osmotic stressors [52,53,54]. The Δ*Cfsec22* mutant’s sensitivity to multiple stressors and abnormal chitin synthase and ER chaperone expression patterns further support this conserved role of vesicular transport in stress adaptation. As known to dynamically respond to extracellular conditions, the vacuolar system may be particularly affected by CfSec22 deficiency, leading to defective stress responses. The particular importance of these mechanisms during host infection becomes evident when considering the substantial osmotic pressures pathogens encounter within plant cells rich in solutes and salt ions.

Reactive oxygen species (ROS) play pivotal roles in pathogen–host interaction signaling pathways. Fungal NADPH oxidases (NOX1 and NOX2) regulate ROS accumulation [55,56], which enhances invasion capability in *M. oryzae* [57,58]. Notably, disruption of vesicular transport components like MoVam7 or MoSec22 impairs ROS accumulation and pathogenicity [28,59]. Our observation of significantly diminished ROS accumulation in Δ*Cfsec22* hyphal tips suggests a similar disruption of redox signaling may contribute to the mutant’s reduced virulence. This connection between vesicular transport, redox homeostasis, and pathogenic capacity appears to be conserved across fungal pathogens.

Sweet potato responds to *C. fimbriata* infection by producing furanoid toxins (primarily ipomeamarone) [15]. Intriguingly, ipomeamarone was undetectable in the Δ*Cfsec22*-infected sweet potato, suggesting ipomeamarone may be produced by sweet potato in response to certain elicitors secreted by *C. fimbriata*. The ER localization of CfSec22 and its role in vesicle trafficking suggest that defective secretion of these elicitor molecules underlies this phenotype. In this study, the absence of *CfSEC22* leads to defective endocytosis and alterations in the secretory profile. Since endocytosis is a crucial factor in maintaining a balance between vesicular loss and recovery during vesicular transport, CfSec22’s absence likely disrupts secretion of infection-associated proteins via ER–Golgi pathways, endocytic vesicle recovery, and overall secretory profile. While the small lesion size caused by the mutant may contribute to reduced toxin production, the more fundamental disruption appears to be in the secretory pathway itself.

Secreted proteins are crucial for fungal pathogenesis, with their transport tightly regulated by vesicle trafficking proteins [29,60]. Our proteomic analysis revealed extensive alterations in the secretory profile of Δ*Cfsec22*, with 223 proteins showing significant downregulation, including confirmed extracellular proteins, hydrolases, carbon–nitrogen lyases, and multiple redox regulators. Notably, 90% of the top 10 downregulated extracellular proteins contained functional signal peptides, implicating CfSec22 in conventional secretory pathways. These secretion defects likely explain the mutant’s attenuated virulence, as similar deficiencies in other fungal systems consistently impair the secretion of key virulence factors. In *M. oryzae*, MoSyn8 regulates the secretion of non-toxic effectors, such as Avr-Pia and AvrPiz-t, thereby modulating the interaction between *M. oryzae* and rice [48]. MoSso1 regulates pathogenicity by participating in the secretion of cytoplasmic effector [61]. The *Sclerotinia sclerotiorum* mutants Δ*Ssemp24* and Δ*Sserv25* show parallel reductions in hydrolase secretion and virulence [62]. In *V. dahliae*, VdSec22 and VdSso1 mediate extracellular protein transport and virulence [29]. Our findings extend this pattern to *C. fimbriata*, where CfSec22-mediated transport appears crucial for delivering a broad spectrum of pathogenic determinants.

Among the downregulated proteins, several merit particular attention due to their established roles in fungal pathogenesis. The subtilisin-like proteinase Spm1 (PHH54118.1), previously implicated in autophagy processes, represents one such factor [63]. Similarly, the pectin lyase (PHH55093.1) contributes to host cell wall degradation [64], while glutathione (PHH55903.1) maintains cellular redox balance [65]. The collective reduction in such hydrolytic enzymes likely impairs host tissue degradation, while diminished effector secretion would compromise the pathogen’s ability to counteract host defenses. This dual deficiency provides a plausible explanation for the limited lesion expansion observed in infection assays, though the specific contributions of individual secreted proteins remain to be fully elucidated.

## 5. Conclusions

Through comprehensive characterization of CfSec22 in *Ceratocystis fimbriata*, this study has revealed the multifaceted roles of this conserved vesicular transport component. Beyond its expected functions in growth and sporulation, CfSec22 emerges as a critical regulator of stress responses, maintaining proper vacuolar morphology and endocytic processes. Its ER localization and role in protein secretion position it as a central coordinator of pathogenic processes, from regulation of ROS balance to control of secretome composition and ipomeamarone induction. The convergence of these defects in the Δ*Cfsec22* mutant underscores the integrative nature of vesicular transport in fungal pathogenesis, where proper membrane trafficking supports virtually all aspects of host interaction and virulence. Collectively, our findings establish that CfSec22-mediated vesicular transport critically supports *C. fimbriata* development and pathogenesis through multiple mechanisms.

## Figures and Tables

**Table 1:** Analysis of significantly downregulated extracellular protein information (* *p* < 0.05, ** *p* < 0.01, and *** *p* < 0.001).

Protein Locus	Description	Unique Peptides	Signal Peptide	COVERAGE (%)	Mutant/Wild-Type	Subcellular Loc
PHH53199.1	Glucan 1,3-beta-glucosidase	17	Y	24.7	0.02938 ***	extracellular
PHH54118.1	Subtilisin-like proteinase Spm1	11	Y	23.9	0.4911 ***	extracellular
PHH50969.1	Endoglucanase EG-II	9	Y	24.1	0.3528 **	extracellular
PHH54020.1	Kre9_KNH domain-containing protein	4	Y	17.8	0.2825 **	extracellular
PHH51195.1	Lysophospholipase	8	Y	18.6	0.4424 ***	extracellular
PHH54561.1	Glucan 1,3-beta-glucosidase	11	Y	17.6	0.02668 ***	extracellular
PHH55093.1	Putative pectin lyase A	5	Y	16	0.2716 **	extracellular
PHH55903.1	Glutathione hydrolase	7	Y	18.7	0.6418 *	extracellular
PHH54624.1	alpha-amylase	7	N	16.5	0.4479 **	extracellular
PHH54693.1	Putative endo-beta-1,4-glucanase B	3	Y	8.9	0.3617 *	extracellular

## Data Availability

The original contributions presented in this study are included in the article/supplementary material.

## Supplementary Materials

The following supporting information can be downloaded at: https://www.mdpi.com/article/10.3390/microorganisms13102305/s1, Table S1: Sequences of the primers used in this study; Figure S1: Analysis of CfSec22 characteristics; Figure S2: *CfSEC22* knockout and mutation experience; Figure S3: Internal inspection and external inspection. Figure S4: Phylogenetic analysis of Spm1 from typical species; Figure S5: Phylogenetic analysis of Gsh from typical species. The proteome data reported in this paper have been deposited in the OMIX, China National Center for Bioinformation/Beijing Institute of Genomics, Chinese Academy of Sciences (https://ngdc.cncb.ac.cn/omix: accession no.OMIX010249, accessed on 1 May 2025).
