# Supplementary material for: SNARE Protein CfSec22 Mediates Vesicular Trafficking to Regulate Growth, Conidiogenesis, and Pathogenesis of Ceratocystis fimbriata
Source: Microorganisms. 2025 Oct 5;13(10):2305. doi: 10.3390/microorganisms13102305 (PMC12566070; doi:10.3390/microorganisms13102305)
Supplement: Supplementary file 1 [file microorganisms-13-02305-s001.zip › microorganisms-3832680-supplementary.pdf]

Table S1. Sequences of the primers used in this study.

Primer name/ Purpose	Primer sequence (5'-3')
SEC22-1F	AGATTTCGTCTGTGGGCGTT
SEC22-2R	TTGACCTCCACTAGCTCCAGCCAAGCCGGCCAAGACTAATAGGCGCA
SEC22-3F	CAAAGGAATAGAGTAGATGCCGACCG GGATTGCCCGTTTTTGCCTT
SEC22-4R	AGCCGTTTGCTACGGAAGAA
SEC22-5F	TCAACGCAAATATCCCGCCT
SEC22-6R	CAAGTTTGCGCGGGTATGAG
SEC22-7F	GACCGCGCGATAGACTATGA
SEC22-9F	ACTCACTATAGGGCGAATTGGGTACTCAAATTGGTTTTGACGTTCACATCGACACC
SEC22-10R	CACCACCCCGGTGAACAGCTCCTCGCCCTTGCTCACAAAGAACCGGAGATAGATAAACAAC
HYGF	GGCTTGGCTGGAGCTAGTGGAGGTCAA
HYR	TATTGACCGATTCCTTGCGGTCCGAA
YGF	GATGTAGGAGGGCGTGGATATGTCCT
HYGR	CGGTCGGCATCTACTCTATTCCTTTG
First round of overlap PCR	(1F, 2R); (3F, 4R); (HYGF, HYR); (YGF, HYGR)
Second round of overlap PCR	(1F, HYR); (YGF, 4R)
Third round of overlap PCR	(1F, 4R)
Complementary fragment	(9F, 10R)
Validation of yeast plasmids	(5F, 6R)
Internal verification of PCR	(5F, 6R)
External verification of PCR	(7F, HYGR)
Probe 1 of Southern blot	(5F, 6R)
Probe 2 of Southern blot	(HYGF, HYR)
LHS1-11	AGCAAGCTCCCCTCAACATC
LHS1-12	ACGTGGACTCTGTCAAGCAG
KAR2-11	AGGAGCCCAACAAGAGCATC
KAR2-12	GCAGGATCTCGCTAGTGGTC
SCJ1-11	ATCGCCAACTTAGCAAACGC
SCJ1-12	TCTTTCGGGTTTCAGGGTCG
SIL1-11	GGCCAATCTAGGCCTTTGGT
SIL1-12	CTAGCATGCGTTCCTGGCTA
ACTIN-1f	GTCACTCACGTCGTTCCCAT

ACTIN-2r	CACGCTCGGCAGTAGTAGAG
COM1-1f	TCCAGTCAGCGTGAAAGCAT
COM1-2r	GCCAAGGACCAACGTGTTTC
HOX2-1r	ATGAGCCCCAACACCCAAAA
HOX2-2f	CGAAACGCTAGCATCTGTGC
COS1-1f	TCCCAACGCCAATTCGAAGA
COS1-2r	TCCTCGGGCACTTGAATAGC
CON7-1f	GGACACCAGCTACATTCCGT
CON7-2r	CCGGGTCCGCTTATATTCCC
STUA-1f	AGCGCAAGGTACACACTCAA
STUA-2r	GGAGGGCGAGAGTCGTAATG
CON2-1f	CGTGACCTGGATAAACGCCT
CON2-2r	CCCTTGCTCTTTCCACGAGT
CHS1-1f	TTGGTAATCGTCCCAAGGGC
CHS1-2r	TTCAGGGTCGACATTGCGAA
CHS2-1f	TTGTCGTCTGCATCGTGTCA
CHS2-2r	CGGCCTTTTGGTTCACGAAG
CHS3-1f	AATCCGCTACTGGCGAAACA
CHS3-2r	GTGGTCGGAACGGAAGAAGT
CHS4-1f	TACGTGGCTGTTGCTAGTGG
CHS4-2r	GGTGTGCCGCATTTCACAAT
CHS6-1f	GGGTCGCTATTGTTTGGGGA
CHS6-2r	ACGAACTTTTCACGCCAAGC
CHS8-1f	ACGGGCATTAACGGGAACAT
CHS8-2r	ATTGGTCCATGCACGGAAGT
SPM1-1F	GATCTGTTTTCCGCAAGGGC
SPM1-2R	TTGACCTCCACTAGCTCCAGCCAAGCC TTCGCAAATGATGACGGCAG
SPM1-3F	CAAAGGAATAGAGTAGATGCCGACCG TGGCTCTCGGGAGTATAGGG
SPM1-4R	AGGAATCAAACGGCACCCAA
SPM1-5F	GAGACGGAGAAGAACTCGCC
SPM1-6R	CTTGCCCCAGTTGGAGAAGT
SPM1-7F	GATGATGGTACTTCCTGTAGTC
GSH-1F	CAAGTTACGAGCGAGAGCCA
GSH-2R	TTGACCTCCACTAGCTCCAGCCAAGCC TGTGGTGAGCAGAGGGAGAT
GSH-3F	CAAAGGAATAGAGTAGATGCCGACCG CCTGGATTTCTTTGCAGCGGT
GSH-4R	CCTCATCTCAAGGACCGATGG
GSH-5F	GTTGTGTCCAGAGACCTGCAG
GSH-6R	TGGTACACACAACCGCGAGC
GSH-7F	ACGTCATGAACACTCCTGCACG

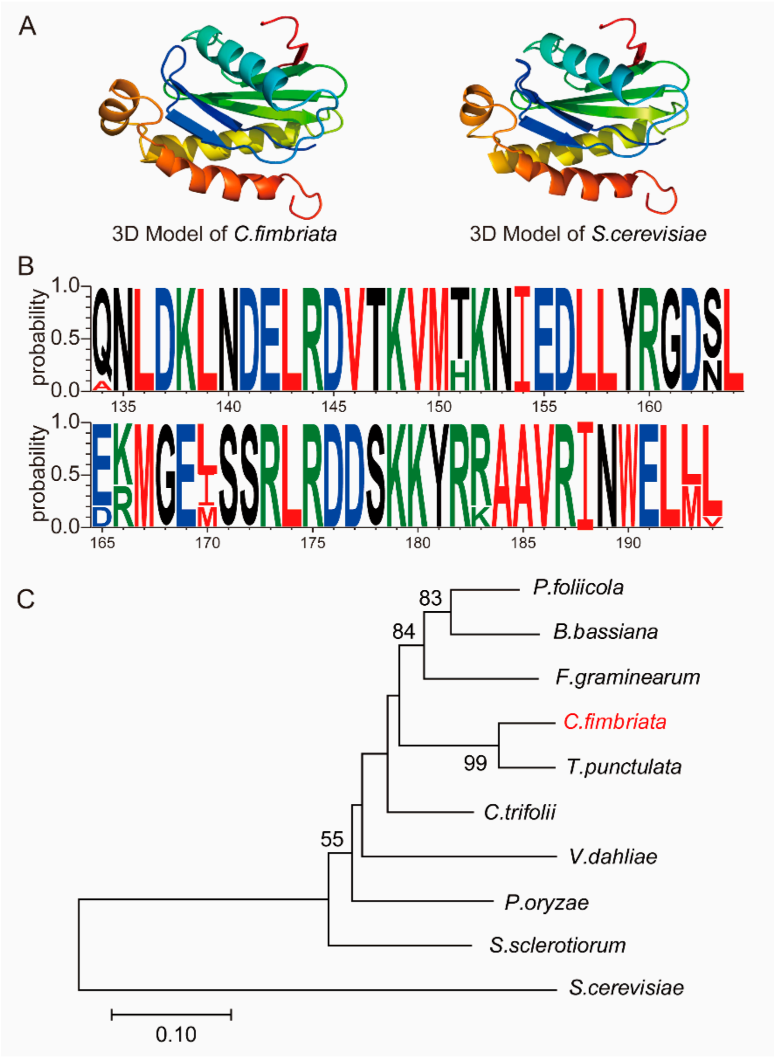

Figure S1. Analysis of CfSec22 characteristics. (A) Sec22 3D structure prediction. (B) Logo map of V-SNARE cooked coil homology region of CfSec22 protein (green represents KRH, blue represents DE, red represents AVLIPWFM, and black represents other amino acids). (C) Phylogenetic analysis of Sec22 protein in typical species.

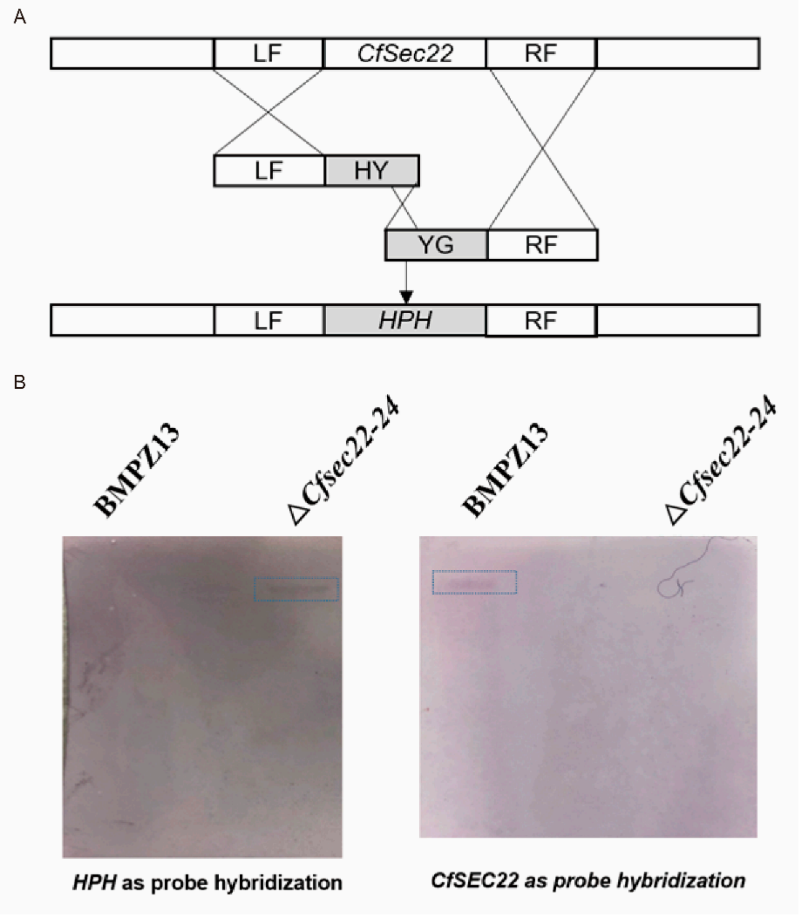

Figure S2. *CfSEC22* knockout and mutation experience. (A) Illustration of the CfSec22 targeted gene replacement strategy. (B) Mutant transformants were verified by Southern blotting analysis.

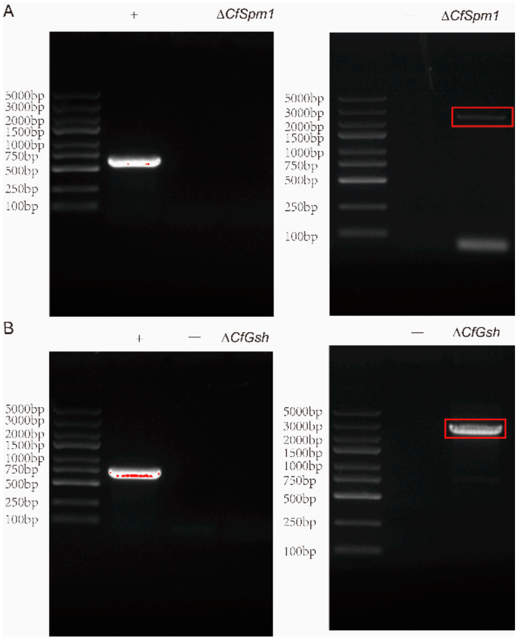

Figure S3. Internal inspection and external inspection

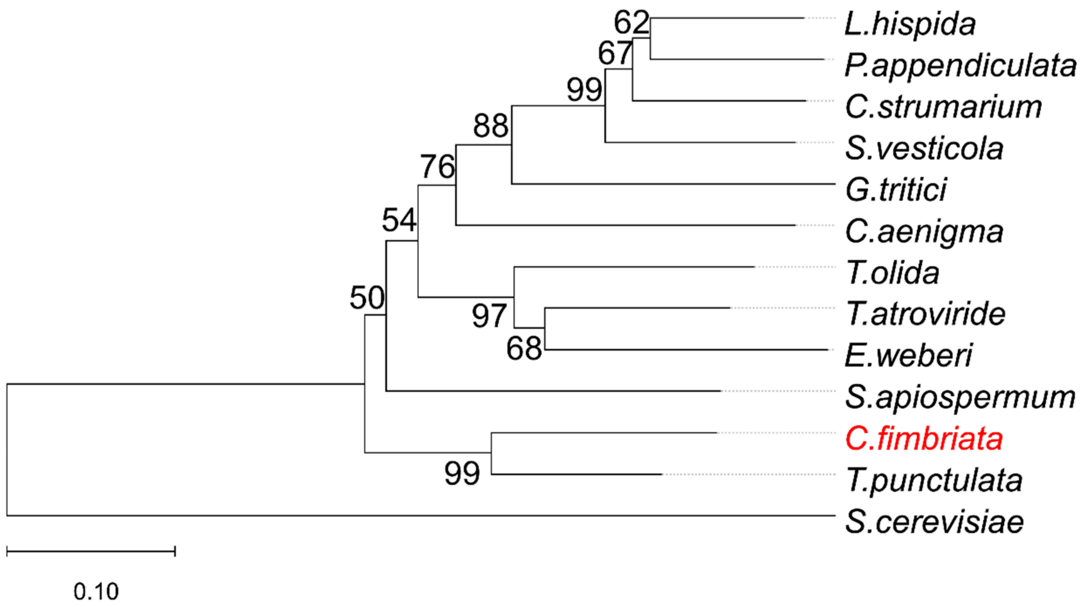

Figure S4. Phylogenetic analysis of Spm1 from typical species. Numbers near the nodes stand for bootstrap values (only values > 50 % are shown).

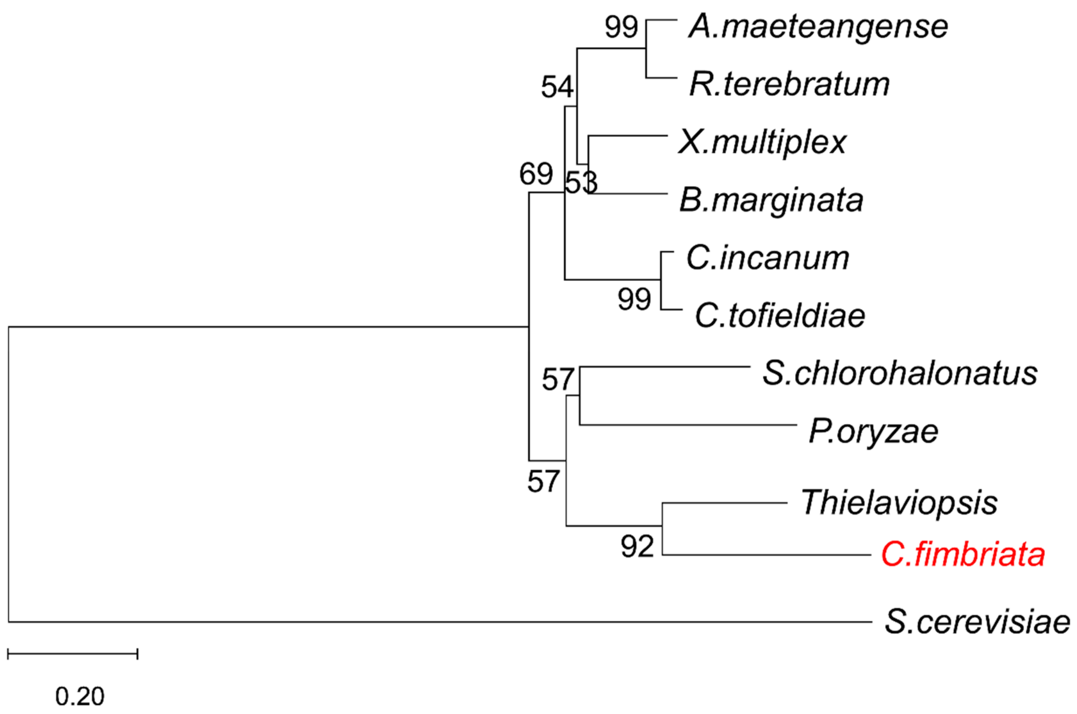

Figure S5. Phylogenetic analysis of Gsh from typical species. Numbers near the nodes stand for bootstrap values (only values > 50 % are shown).
